# Comparatively sonophotochemical and photochemical studies of phthalocyanines with cationic substituents on nonperipheral positions

**DOI:** 10.55730/1300-0527.3602

**Published:** 2023-09-26

**Authors:** Mukaddes ÖZÇEŞMECİ, Ceren CAN KARANLIK, Ali ERDOĞMUŞ, Esin HAMURYUDAN

**Affiliations:** 1 Department of Chemistry, Faculty of Science and Letters, Istanbul Technical University, İstanbul, Turkiye; 2 Department of Chemistry, Faculty of Science and Letters, Yıldız Technical University, İstanbul, Turkiye

**Keywords:** Photodynamic therapy, phthalocyanine, singlet oxygen, sonophotodynamic therapy

## Abstract

The term sonophotodynamic therapy (SPDT) refers to a combination of sonodynamic therapy (SDT) and photodynamic therapy (PDT), in which the efficacy of the treatment is boosted by utilizing the proper amount of a sensitizer that is responsive to both light and ultrasound. Although it has been proven in photophysicochemical studies that SPDT enhances singlet oxygen production, related studies in the literature are very limited. Considering this situation, this study aims to investigate the efficacy of synthesized phthalocyanines in terms of PDT and SPDT. The singlet oxygen quantum values calculated as 0.13 for **5**, 0.44 for **6**, and 0.61 for **7** in photochemical (PDT) application increased to 0.18, 0.86, and 0.92, respectively, with sonophotochemical (SPDT) application. According to the results, singlet oxygen production was more efficient with SPDT. This work will add to the body of knowledge on employing the SPDT approach to increase singlet oxygen generation.

## 1. Introduction

Cancer is one of the diseases that negatively affect human health worldwide [1,2]. Great success has been achieved with traditional clinical treatments of cancer, such as surgical treatment, chemotherapy, and radiotherapy, and these are still being applied [3,4]. However, these treatment methods have many disadvantages as long-term side effects, toxicity, drug resistance, and low selectivity. For this reason, improving new treatment methods that reduce the death rate and increase the patient’s quality of life in the fight against cancer is very important [3,5].

A potential therapy called photodynamic therapy (PDT) combines three elements: light, a medication called a photosensitizer, and molecular oxygen that damages tumor cells by photodamage [6–10]. Although this approach has several benefits, its effectiveness in treating deeply positioned cancers has been constrained by low light penetration into tissue layers [5,11]. Recently, a new and promising method, known as sonodynamic therapy (SDT), derived from PDT has emerged [12–15]. The main difference between the two methods is activating the sensitizers. In SDT, the sensitizer is activated by ultrasound rather than by light [16,17]. However, this method requires high concentrations of sensitizers, which can result in toxicity [5,18]. In order to overcome all these problems, a new approach called sonophotodynamic therapy (SPDT) is derived by combining PDT and SDT methods [19–23]. The sensitizers selected in SPDT are nontoxic structures with high sonophotosensitivity, they have stable chemical composition, low aggregation tendency, and good solubility properties [5,16,24].

Phthalocyanines (Pcs) are aromatic macrocycles that have undergone substantial research due to their distinctive characteristics and wide range of uses in biology, medicine, and materials [25–38]. Pcs are among the promising second- and third-generation photosensitizers due to their high absorption in the phototherapeutic window and their efficiency in reactive oxygen generation. Also, diamagnetic central metal ions (such as Zn^2+^, In^3+^, and Ga^3+^) containing Pcs are used as photosensitizers due to their efficiency in reactive oxygen generation and high singlet oxygen quantum yields.

The low solubility and high aggregation of Pcs in common organic solvents are a disadvantage for many applications. Especially water solubility is important in biological and medical applications [39]. As a result, it is necessary to construct Pcs with ionic or nonionic substituents in peripheral, nonperipheral, or axial places. Cationic Pcs are obtained with quaternization of aliphatic or aromatic nitrogen atoms in the molecule [27,30,31,40–42]. We have quaternized 1,3-bis-(trimethylamino)-2-propoxy groups where Pcs are substituted with methyl iodide, leading to their water-soluble derivatives. So an important result, namely water solubility, has been provided. Comparatively to metal-free Pc, the photophysical, photochemical, and sonophotochemical characteristics of the produced metallo-Pc derivatives (M: Zn(II) and In(III)Cl) were examined.

## 2. Results and discussion

### 2.1. Synthesis and characterization

**Figure 1:**
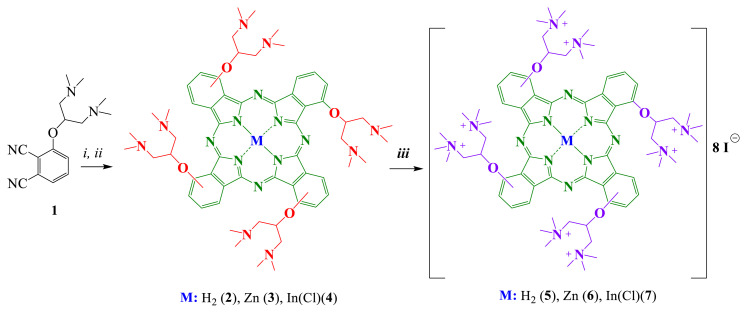
Synthesis of phthalocyanines (**2–7**) (*i*: lithium metal, *n*-pentanol, 145 °C, acetic acid; *ii*: metal salt, 2-dimethylaminoethanol, 140 °C, 24 h, *iii:* CH_3_I, CHCl_3_, at dark, reflux temperature).

The synthetic processes for producing the metal-free Pc and metallo-Pc derivatives (**2**–**7**) are shown in Figure 1. Compound **1** was prepared according to the reported procedure [43]. Compound 1 and lithium metal were combined in n-pentanol for the production of the metal-free Pc derivative (**2**), which was subsequently acidified with 1 mL of glacial acetic acid. The indium(III) Pc derivative (**4**) was synthesized by the addition of InCl_3_ to compound **2** in 2-dimethylaminoethanol. UV-Vis spectra obtained in 2-dimethylaminoethanol showed the elimination of the Q band of metal-free Pc and the emergence of a new Q band of indium Pc. Water soluble cationic Pcs **5** and **7** were obtained with quaternization of aliphatic nitrogen atoms in compounds **2** and **4** with methyl iodine. Pcs **3** and **6** were prepared using the synthetic procedure described in Ref. [27].

In this study, all synthesized compounds were characterized using several spectroscopic methods. Each compound’s spectral information matched the formulations.

The FT-IR spectra of **2** and **4** showed no strong nitrile vibrations at 2229 cm^−^^1^, which supported the production of Pc derivatives. Synthesized Pcs’ FT-IR spectra were similar, except for the metal-free derivatives (**2** and **5**). They displayed additional absorption bands attributed to the -NH stretching vibrations at 3291 cm^−^^1^. Broad peaks were observed in the ^1^H NMR spectra in DMSO-d_6_ due to the mixed structural isomers of tetrasubstituted Pcs. The multiplets of aromatic protons of Pcs were seen at 8.28–7.40 (**2**), 9.22–7.78 (**4**), 9.11–7.49 (**5**), and 9.41–8.17 (**7**) ppm, respectively. The OCH protons were observed as multiplets at 5.30 (**2**), 5.12 (**4**), 6.27 (**5**), and 6.16 (**7**) ppm, respectively. The NCH_2_ protons were observed at 3.81 (**2**), 2.83 (**4**), 4.63 (**5**), and 4.19 (**7**) ppm, respectively. Also, −NCH_3_ protons were seen at 3.11 (**2**), 2.42 (**4**), 3.09 (**5**), and 3.43 (**7**) ppm, respectively. The inner core protons were also monitored with broad chemical shifts at −1.38 (for **2)** and −1.40 (for **5**) ppm.

The molecular peaks at m/z 1091.192 [M]^+^ (for **2**), 1239.159 [M]^+^ (for **4**), 1338.573 [M - 7I]^+^, 1211.111 [M - 8I]^+^ (for **5**), 1740.699 [M - 5I]^+^, 1360.171 [M - 8I]^+^ (for **7**) were seen in the MALDI-TOF mass spectra.

**Figure 2:**
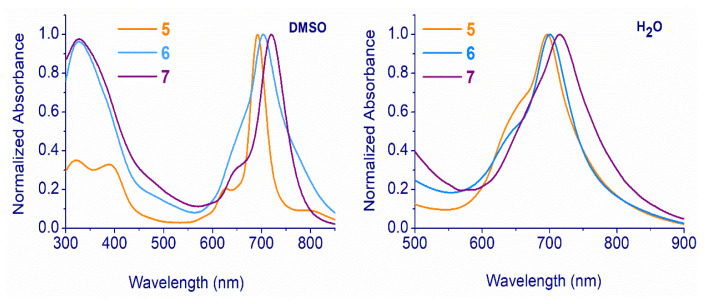
UV-Vis absorption spectra of **5**–**7** in DMSO (**a**) and water (**b**) at 1 × 10^−^^5^ M.

Two prominent peaks can be seen in the electronic absorption spectra of Pcs **2** and **4** in THF: the typical ligand-centered π–π* transitions of a monomeric metal-free (**2**) and indium(III) (**4**) Pcs with Q-band maxima at 694 and 726 (for **2**) and 717 (for **4**) nm. Due to its D*_2h_* symmetry, the Q band of the metal-free derivative (**2**) showed signs of splitting [44]. The additional Pcs-specific bands, or B bands, were seen at 319 and 322 nm for Pcs **2** and **4**, respectively. Indium(III) phthalocyanine (**4**) possesses Q bands that change bathochromically at 17 nm when compared to the λ_max_ value of the zinc(II) phthalocyanine (**3**) [27]. This can be explained by the fact that indium, as the central metal ion, remains outside the cavity due to its larger radius. Quaternized derivatives of compounds **5** and **7** showed B bands in DMSO at 322 and 335 nm, respectively. In DMSO, the Q bands were recorded at 693 (for **5**) and 720 nm (for **7**). In addition, the absorption spectra of quaternized Pcs **5** and **7** in water displayed B bands at 319 (for **5**) and 335 (for **7**) nm and Q bands at 693 (for **5**) and 715 (for **7**) nm (Figure 2).

### 2.2. Photophysical and photochemical studies

#### 2.2.1. Fluorescence quantum yield (Φ_F_)

**Figure 3:**
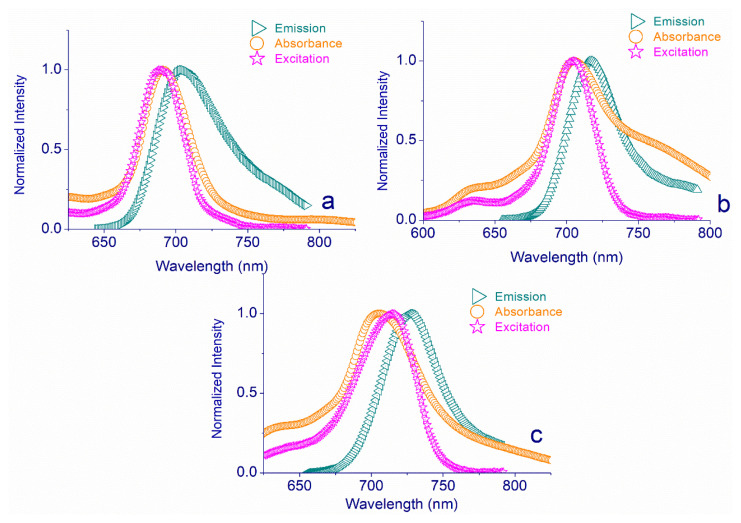
**a)** Absorption (693 nm), excitation (693 nm), and emission (704 nm) spectra of **5**, **b)** absorption (699 nm), excitation (699 nm), and emission (717 nm) spectra of **6**, **c)** absorption (720 nm), excitation (722 nm), and emission (727 nm) spectra of **7**.

Fluorescence characteristics of molecules enable traceability of their in biological systems in PDT applications. Here, the fluorescence characteristics of **5**, **6**, and **7** were examined in DMSO, and they displayed emission bands at 705 nm with fluorescence quantum yield (Φ_F_) of 0.05 (for **5**), at 717 nm with Φ_F_ of 0.056 (for **6**) and 727 nm with Φ_F_ of 0.030 (for **7**). The results were recorded in Table, and Figure 3 depicts the obtained spectral changes (absorption, emission, and excitation).

In DMSO, unsubstituted ZnPc has a greater fluorescence quantum yield (Φ_F_: 0.20) [45] than Pcs (**5**–**7**). The presence of heavy atoms such as indium in the central cavity of Pcs contributes to ISC, resulting in high singlet oxygen quantum yield and low fluorescence quantum yield. The fluorescence quantum yield of unsubstituted indium(III) Pc (CIInPc) was calculated as 0.018 [46] in DMSO. A slight increase in Φ_F_ value of compound **7** was observed compared to CIInPc.

#### 2.2.2. Singlet oxygen quantum yields (Φ_Δ_)

When excited with a specific wavelength of light or ultrasound frequency, sensitizers can change molecular oxygen into reactive oxygen species. Singlet oxygen, one of these reactive oxygen species, constitutes one of the fundamental components of PDT. Although the sensitizer directly influences the formation of singlet oxygen, the sensitizer’s stimulation technique has a significant impact as well. In the stimulation of sensitizers, suitable wavelengths of light, ultrasound, and light/ultrasound can be used together. We describe it as “photodynamic therapy” when light is used as a stimulation method, “sonodynamic therapy” when sound waves are used, and “sonophotodynamic therapy” when ultrasound and light are used together [24]. Studies in the literature have shown that the SPDT approach increases singlet oxygen generation [13,17,45,47,48]. Using photodynamic and sonophotodynamic techniques, we investigated the relationship between singlet oxygen generation and irradiation method to evaluate the singlet oxygen-producing capacities of the cationic Pcs (**5**–**7**). The singlet oxygen quantum yields of cationic Pcs (**5**–**7**) were calculated for both methods using unsubstituted ZnPc as standard and 1,3-diphenylisobenzofuran (DPBF) as a chemical singlet oxygen quencher [49]. Pcs (**5**–**7**), standard ZnPc, and DPBF solutions were dissolved in DMSO and kept in a dim environment. In PDT and SPDT applications, the importance of water solubility stands out. However, most Pcs are insoluble or have serious aggregation behavior in water. Because of this, DMSO, a nontoxic solvent for biological cell investigations at the necessary concentration for PDT/SPDT application, has been chosen in related photophysical and photochemical studies [50,51].

**Figure 4:**
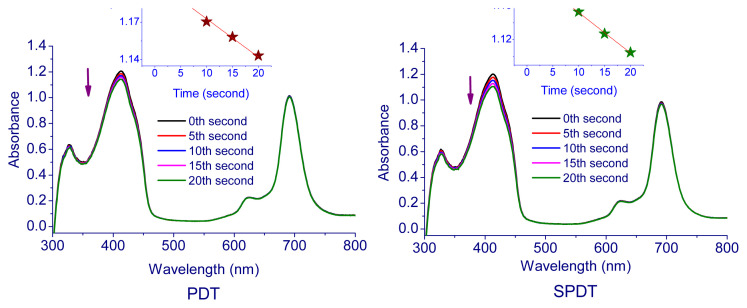
Representative absorption spectral changes during the determination of the singlet oxygen quantum yield of **5**.

**Figure 5:**
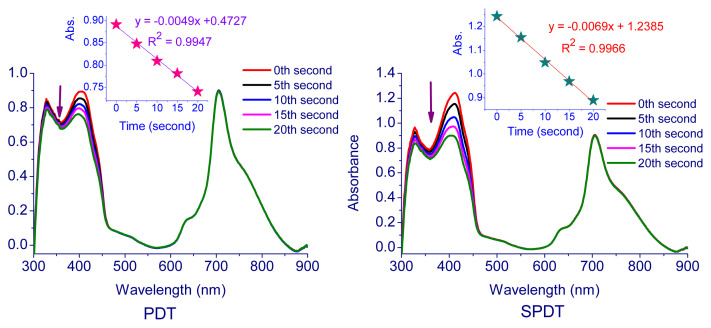
Representative absorption spectral changes during the determination of the singlet oxygen quantum yield of **6**.

**Figure 6:**
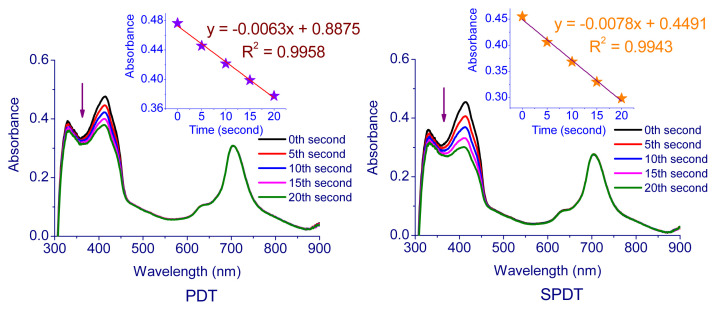
Representative absorption spectral changes during the determination of the singlet oxygen quantum yield of **7**.

In the measurements performed using the photochemical method, the solutions containing Pcs (**5**–**7**) and DPBF were exposed to light (intensity of 7.05 × 10^15^ photons s^−1^ cm^−2^) every 5 s and the change in absorbance of DPBF at 417 nm was followed by using UV-Vis spectrophotometer. Table contains the singlet oxygen quantum yields of compounds **5**–**7**. The corresponding spectra and plot of the DPBF absorption reduction with time are displayed in Figures 4–6. The singlet oxygen quantum yields were calculated as 0.13 for **5**, 0.44 for **6**, and 0.61 for **7**. The results demonstrate that all cationic Pcs (**5**–**7**) promote the intersystem crossing (ISC) more strongly than unsubstituted ZnPc, boosting singlet oxygen generation while lowering fluorescence efficiency. The metal atom in the center is important for increasing the efficiency of the Pc molecule as a sensitizer. A heavy central atom such as indium provides a high singlet oxygen yield because it contributes to ISC. Therefore, indium Pcs can have high singlet oxygen quantum yields [52,53].

In studies carried out with the photosonochemical method, the solutions were first exposed to light for 5 s and to ultrasound (35 kHz) every 5 s, and a UV-Vis spectrophotometer was used to monitor the change in DPBF’s absorbance at 417 nm. The SPDT approach yielded singlet oxygen quantum efficiencies of 0.18 for **5**, 0.86 for **6**, and 0.92 for **7**. It was observed that SPDT application increased the singlet oxygen formation considerably compared to PDT only. The mechanism of activating the sensitizer by the SDT method is not yet fully understood, but possible processes have been described [54]. In a study in which only light, only sound and light + sound were used together [55], the use of light + sound increased the singlet oxygen generating capacity more than the methods in which only light and only sound were used. Also, it has been observed that the method in which only light is used as a stimulant is more effective than the method in which only sound is used as a stimulant. In our different studies, we examined the effect of the stimulant on singlet oxygen production as photochemically and sonophotochemically and observed that singlet oxygen production increased significantly when light and ultrasound were used together [56,57]. It has also been reported that the ultrasound effect contributes to the formation of reactive oxygen species such as singlet oxygen. In this way, it was also stated that SPDT technique causes more deaths on various cancer cells than PDT [4, 58, 55].

#### 2.2.3. Photodegradation quantum yields *(*Φ_d_*)*

**Figure 7:**
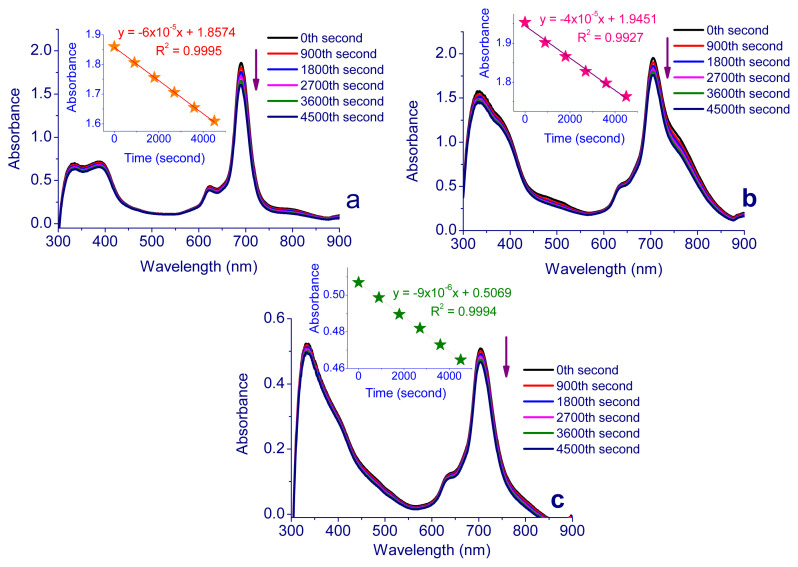
Representative absorption spectral changes during the determination of the photodegradation quantum yield of Pcs **a)** for **5**, **b)** for **6**, and **c)** for **7**.

High singlet oxygen production, fluorescence, solubility in water or biocompatible solvents to accumulate in tumor tissue and photostability are expected properties of sensitizers for PDT and SPDT applications. Singlet oxygen production starts with the stimulation of the sensitizer, so it is very important for the treatment to keep the concentration of the sensitizer constant during the treatment. For this reason, the photostabilities of Pcs (**5**–**7**) were calculated by utilizing the change in the absorption band by exposing them to 5 min of photoirradiation via UV-Vis spectroscopy in the biocompatible solvent DMSO. Table contains the related data, while Figure 7 shows the spectra. The result showed that Pcs were stable during photodegradation. F_d_ values of the Pcs are 8.7 × 10^−^^4^ for **5**, 1.2 × 10^−^^4^ for **6**, and 1.4 × 10^−^^4^ for **7**. Obtained results are compared with the literature, and the photostability of Pcs agrees with those of other studies [59].

## 3. Conclusion

In this study, metal-free and metallophthalocyanines having 1,3-bis-(trimethylamino)-2-propoxy groups at the nonperipheral positions were synthesized and characterized. In addition, the capability of the Pcs to generate singlet oxygen was studied photochemically and sonophotochemically. In photochemical measurements, the singlet oxygen values were 0.13 for **5**, 0.44 for **6**, and 0.61 for **7**. In sonophotochemical measurements, the singlet oxygen values increased to 0.18 for **5**, 0.86 for **6**, and 0.92 for **7**. Based on the singlet oxygen quantum values formed by the different irradiation methods of the Pcs and their photostability during these application methods, especially metallo derivatives can be considered to be good sensitizing agents in PDT and SPDT applications.

## Supplementary Information

**Comparative sonophotochemical and photochemical studies of phthalocyanines with cationic substituents on nonperipheral positions**

## 1. Materials and equipment

All reagents and solvents were of reagent grade quality and were obtained from commercial suppliers. ^1^H NMR spectra of the synthesized compounds were recorded on an Agilent VNMRS 500 MHz spectrometer. FT-IR spectra were recorded on a PerkinElmer Spectrum One FT-IR spectrometer with a UATR (universal attenuated total reflectance sampling accessory) module. Electronic spectra were recorded on a Scinco SD 1000 diode array, single-beam ultraviolet–visible (UV–Vis) spectrophotometer. Mass spectra were performed on Bruker Microflex MALDI-TOF mass spectrometer. 3-[1,3-bis(dimethylamino)-2-propoxy]-1,2-dicyanobenzene (**1**), 1,8(11),15(18),22(25)-tetrakis(1,3-bis-(dimethylamino)-2-propoxy)phthalocyaninato zinc(II) (**3**) and [1,8(11),15(18),22(25)-tetrakis(1,3-bis-(trimethylamino)-2-propoxy) phthalo-cyaninatozinc(II)]octaiodide (**6**) were prepared according to the reported procedures [S1,S2].

Fluorescence spectra were measured using a Varian Eclipse spectrofluorometer using 1 cm path length cuvettes at room temperature. Photoirradiations were measured using a General Electric quartz line lamp (75W). A 600 nm glass cut off filter (Schott) and a water filter were used to filter off ultraviolet and infrared radiations respectively. Light intensities were measured with a POWER MAX5100 (Mol electron detector incorporated) power meter. Bandelin Ultrasonic RK 100 H was used for ultrasound irradiation.

## 2. Experimental

### 2.1. Synthesis

#### 2.1.1. 1,8(11),15(18),22(25)-Tetrakis(1,3-bis-(dimethylamino)-2-propoxy) phthalocyanine (2)

0.010 g (1.5 mmol) of lithium metal in small amounts was mixed with 5 mL of n-pentanol and the mixture was stirred at 80 °C for 10 min. Compound **1** was added to the reaction mixture in the amount of 0.200 g (0.734 mmol), and the reaction temperature was raised to 145 °C. For an additional 5 h, the mixture was continued to be stirred in a closed tube under nitrogen atmosphere. The resulting green product was precipitated with cold water after cooling to room temperature. The metal-free phthalocyanine structure was obtained by protonation by adding 1 mL of glacial acetic acid. The product was purified using alumina-packed column chromatography and a THF/methanol (1:9) mixture as the eluent. C_60_H_82_N_16_O_4_; Yield: 0.074 g (37%). m.p. > 200°C. FT-IR ν/cm^−^^1^: 3291 (N-H), 3068 (Ar-H), 2941–2770 (Aliph. C-H) 1584, 1485, 1258, 1019, 746. ^1^H-NMR (DMSO-d_6_, 500 MHz): 8.28–7.40 (m, 12H, Ar-H), 5.30 (br, 4H, OCH), 3.81 (m, 16H, NCH_2_), 3.11 (s, 48H, NCH_3_), −1.38 (br, 2H, NH) ppm. UV-Vis (THF), l_max_ (log ɛ): 319 (4.82), 694 (5.20), 726 (5.19) nm. MS (MALDI-TOF), (*m/z*) calcd. 1091.420 [M]^+^; found: 1091.192 [M]^+^

#### 2.1.2. 1,8(11),15(18),22(25)-Tetrakis(1,3-bis-(dimethylamino)-2-propoxy) phthalocyaninatoindium(III)chloride (4)

0.050 g compound **2** (0.046 mmol) and 0.020 g InCl_3_ (0.092 mmol) were mixed in a closed tube in 2-dimethylaminoethanol (2 mL) and stirred under a nitrogen atmosphere for 20 h at 140 °C. The product was cooled to room temperature and washed with water and methanol. After that, it was purified by alumina packed-column chromatography using a THF/methanol (1:9) mixture as the eluent. C_60_H_80_ClInN_16_O_4_; Yield: 0.020 g (35%). m.p. > 200 °C. FT-IR ν/cm^−^^1^: 2942–2768 (Aliph. C-H), 1607, 1393, 1224, 1089, 829, 702. ^1^H-NMR (DMSO-d_6_, 500 MHz): 9.22–7.78 (m, 12H, Ar-H), 5.12 (m, 4H, OCH), 2.83 (m, 16H, NCH_2_), 2.42 (s, 48H, NCH_3_) ppm. UV-Vis (THF), l_max_ (log ɛ): 322 (5.31), 717 (4.95) nm. MS (MALDI-TOF), (*m/z*) calcd. 1239.68 [M]^+^; found: 1239.159 [M]^+^.

#### 2.1.3. [1,8(11),15(18),22(25)-Tetrakis[1,3-bis-(trimethylamino)2-propoxy) phthalocyanine]octaiodide (5)

0.050 g compound **2** (0.046 mmol) and 0.653 g methyl iodide (0.460 mmol) were refluxed in 30 mL of chloroform for 20 h. Then the reaction solvent was removed under vacuum. The resulting product was washed repeatedly using chloroform, dichloromethane, methanol, ethanol, and ethyl acetate solvents. C_68_H_106_I_8_N_16_O_4_; Yield: 0.063 g (62%). m.p. > 200 °C. FT-IR ν/cm^−^^1^: 3291 (N-H), 3011 (Ar-H), 2941-2831 (Aliph. C-H), 1473, 1214, 1094, 973, 746. ^1^H NMR (DMSO-d_6_, 500 MHz): 9.11–7.49 (br, 12H, Ar-H), 6.27 (br, 4H, OCH), 4.63 (br, 16H, NCH_2_), 3.09 (s, 72H, NCH_3_), −1.40 (br, 2H, NH) ppm. UV-Vis (DMSO), l_max_ (log ɛ): 322 (4.27), 693 (4.55) nm. UV-Vis (H_2_O), l_max_ (log ɛ): 319 (4.19), 693 (4.44) nm. MS (MALDI-TOF), (*m/z*) calcd. 2226.940 [M]^+^, found: 1338.573 [M - 7I]^+^, 1211.111 [M - 8I]^+^.

#### 2.1.4. [1,8(11),15(18),22(25)-Tetrakis[1,3-bis-(trimethylamino)2-propoxy) phthalocyaninatoindium(III)chloride]octaiodide (7)

Compound **7** was synthesized according to the procedure described for **5**. The amounts of reactants used in this synthesis were as follows: Compound **4** (0.037 g, 0.030 mmol), methyl iodide (0.043 g, 0.303 mmol) in chloroform (30 mL). C_68_H_104_I_8_ClInN_16_O_4_; Yield: 0.029 g (41%). m.p. > 200 °C. FT-IR ν/cm^−^^1^: 3009 (Ar-H), 2953-2857 (Aliph. C-H), 1477, 1231, 1124, 1049, 926, 745. ^1^H NMR (DMSO-d_6_, 500 MHz): 9.41–8.17 (br, 12H, Ar-H), 6.16 (br, 4H, OCH), 4.19 (br, 16H, NCH_2_), 3.43 (s, 72H, NCH_3_) ppm. UV-Vis (DMSO), l_max_ (log ɛ): 327 (4.31), 720 (4.47) nm; UV-Vis (H_2_O), l_max_ (log ɛ): 335 (4.47), 715 (4.44) nm. MS (MALDI-TOF), (*m/z*) calcd. 2375.190 [M]^+^, found: 1740.699 [M - 5I]^+^, 1360.171 [M - 8I]^+^.

## 3. Photophysical and photochemical studies

### 3.1. Fluorescence quantum yields (Φ_F_)

Fluorescence quantum yield (Φ_F_) was determined by applying the comparative method (Eq. 1) [S3],


(1) $$\Phi_{\text{F}}=\Phi_{\text{F}(\text{Std})}\frac{\text{F}.{\text{A}}_{\text{Std}}.{\text{n}}^{2}}{{\text{F}}_{\text{Std}}.\text{A}.{\text{n}}_{\text{Std}}^{\text{2}}}$$

where F and F_Std_ are the area under the fluorescence emission curves of the sample and the standard, respectively. A and A_Std_ are the respective absorbances of the samples and standard (Unsubstituted **ZnPc**) at the excitation wavelengths, respectively. *n^2^* and *n^2^*_Std_ are the refractive indices of solvents used for the sample and standard, respectively. Unsubstituted **ZnPc** (Φ_F_ = 0.20 in DMSO) [S3] was used as the standard. Both the samples and standards were excited at the same wavelength.

### 3.2. Singlet oxygen quantum yields (Φ_Δ_)

Singlet oxygen efficiency was determined in the air (no oxygen bubbled) using the relative method (Eq. 2) with unsubstituted ZnPc as reference and 1,3-diphenylisobenzofuran (DPBF) as chemical quencher for singlet oxygen,


(2) $$\Phi_{\Delta }=\Phi_{\Delta }^{\text{Std}}\frac{\text{R}.{\text{I}}_{\text{abs}}^{\text{Std}}}{{\text{R}}^{\text{Std}}.{\text{I}}_{\text{abs}}}$$

where $\Phi_{\Delta }^{\text{Std}}$ is the singlet oxygen quantum yield for the standard ZnPc ( $\Phi_{\Delta }^{\text{Std}}=0.67$ in DMSO) [S2]. R and R_Std_ are the DPBF photobleaching rates in the presence of the respective samples and standard, respectively. I_abs_ and are the rates of light absorption by the sample and standard, respectively. The samples containing DPBF were prepared in the dark and irradiated at the Q band region. The absorption band of the DPBF was reduced by light irradiation (The light intensity of 7.05 × 10^15^ photons s^−^^1^ cm^−^^2^). The degradation of DPBF was monitored using UV-Vis spectroscopy after each 5 s light irradiation at about 417 nm for photochemical studies. For sonophotochemical studies, the sample (the compound+DPBF) was monitored after each 10 s irradiation (5 s by light intensity of 7.05× 10^15^ photons s^−^^1^ cm^−^^2^ and 5 s by ultrasound at a frequency of 35 kHz).

### 3.3. Photodegradation quantum yields (Φ_d_)

Photodegradation quantum yields were determined using Eq. 3,


(3) $$\Phi_{\text{d}}=\frac{({\text{C}}_{0}-{\text{C}}_{\text{t}}).\text{V}.{\text{N}}_{\text{A}}}{{\text{I}}_{\text{abs}}.\text{S}.\text{t}}$$

where “C_0”_ and “C_t_” are the sample concentrations before and after irradiation respectively, “V” is the reaction volume, “N_A_” is the Avogadro’s constant, “S” is the irradiated cell area, “t” is the irradiation time, “I_abs_” is the overlap integral of the radiation source light intensity and the absorption of the sample. A light intensity of 2.42 × 10^16^ photons s^−^^1^ cm^−^^2^ and/or ultrasound at a frequency of 35 kHz was employed to determine photodegradation [S3,S4]. The degradation of max. Q band was monitored after each 10-min irradiation.

Figure S1 FT-IR spectrum of **2**.

Figure S2 FT-IR spectrum of **4**.

Figure S3 FT-IR spectrum of **5**.

Figure S4 FT-IR spectrum of **7**.

Figure S5 ^1^H NMR spectrum of **2** in d_6_-DMSO.

Figure S6 ^1^H NMR spectrum of **4** in d_6_-DMSO.

Figure S7 ^1^H NMR spectrum of **7** in d_6_-DMSO.

Figure S8 MALDI-TOF MS spectrum of **2**.

Figure S9 MALDI-TOF MS spectrum of **4**.

Figure S10 MALDI-TOF MS spectrum of **5**.

Figure S11 MALDI-TOF MS spectrum of **7**.

ReferencesS1
LoPC
ZhaoB
DuanW
FongWP
KoWH

Synthesis and in vitro photodynamic activity of mono-substituted amphiphilic zinc(II) phthalocyaninesBioorganic & Medicinal Chemistry Letters20071741073107710.1016/j.bmcl.2006.11.01717127058S2
ÖzçeşmeciM
Sancar BaşS
AkkurtB
BolkentŞ
HamuryudanE
Synthesis, characterization and staining performance of peripherally and non-peripherally substituted metallo-phthalocyanines bearing 1,3-bis-(trimethylamino)-2-propoxy groupsNew Journal of Chemistry2020447786779410.1039/d0nj01404dS3
[S3] TayfuroğluO
AtmacaGY
ErdoğmuşA
Novel peripherally substituted zinc phthalocyanine: synthesis, characterization, investigation of photophysicochemical properties and theoretical study‎↱Journal of Coordination Chemistry2017703095310910.1080/00958972.2017.1377340S4
TayfuroğluO
KılıçarslanFA
AtmacaGY
ErdoğmuşA
Synthesis, characterization of new phthalocyanines and investigation of photophysical, photochemical properties and theoretical studies‎Journal of Porphyrins and Phthalocyanines20182225026510.1142/S1088424618500281

## Figures and Tables

**Table:** Photophysicochemical properties of complexes (**5**–**6**) in DMSO.

Pcs	λ_Abs_(nm)	λ_Em_(nm)	Δ_Stokes_(nm)	Log ɛ	Φ_F_	Φ_d_ (10^−4^)	Φ_Δ(PDT)_	Φ_Δ(SPDT)_
**5**	693	704	11	4.55	0.05	8.7	0.13^*^	0.18
**6**	699	717	18	4.69	0.056	1.2	0.44^*^	0.86
**7**	720	727	7	4.47	0.030	1.4	0.61^*^	0.92

• Unsubstituted ZnPc (F_D_): 0.67 in DMSO^*^ was used as a standard in the singlet oxygen quantum yield measurements.
