# Supplementary figures and images for: Comparatively sonophotochemical and photochemical studies of phthalocyanines with cationic substituents on nonperipheral positions
Source: Turk J Chem. 2023 Sep 26;47(5):1160–8. doi: 10.55730/1300-0527.3602 (PMC10760847; doi:10.55730/1300-0527.3602)

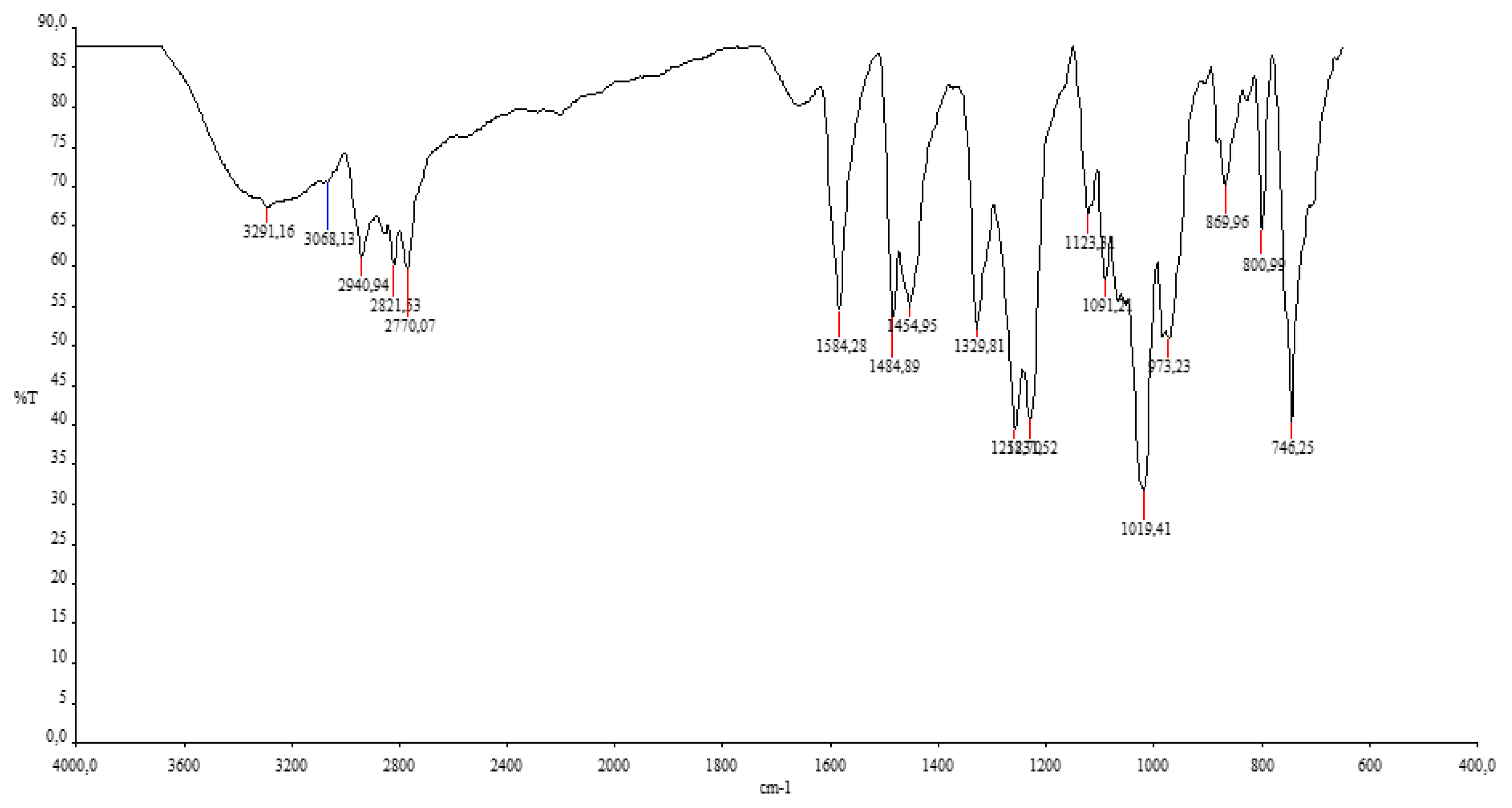

Supplement: Figure S1 — FT-IR spectrum of 2. [file turkjchem-47-5-1160s1.tif]

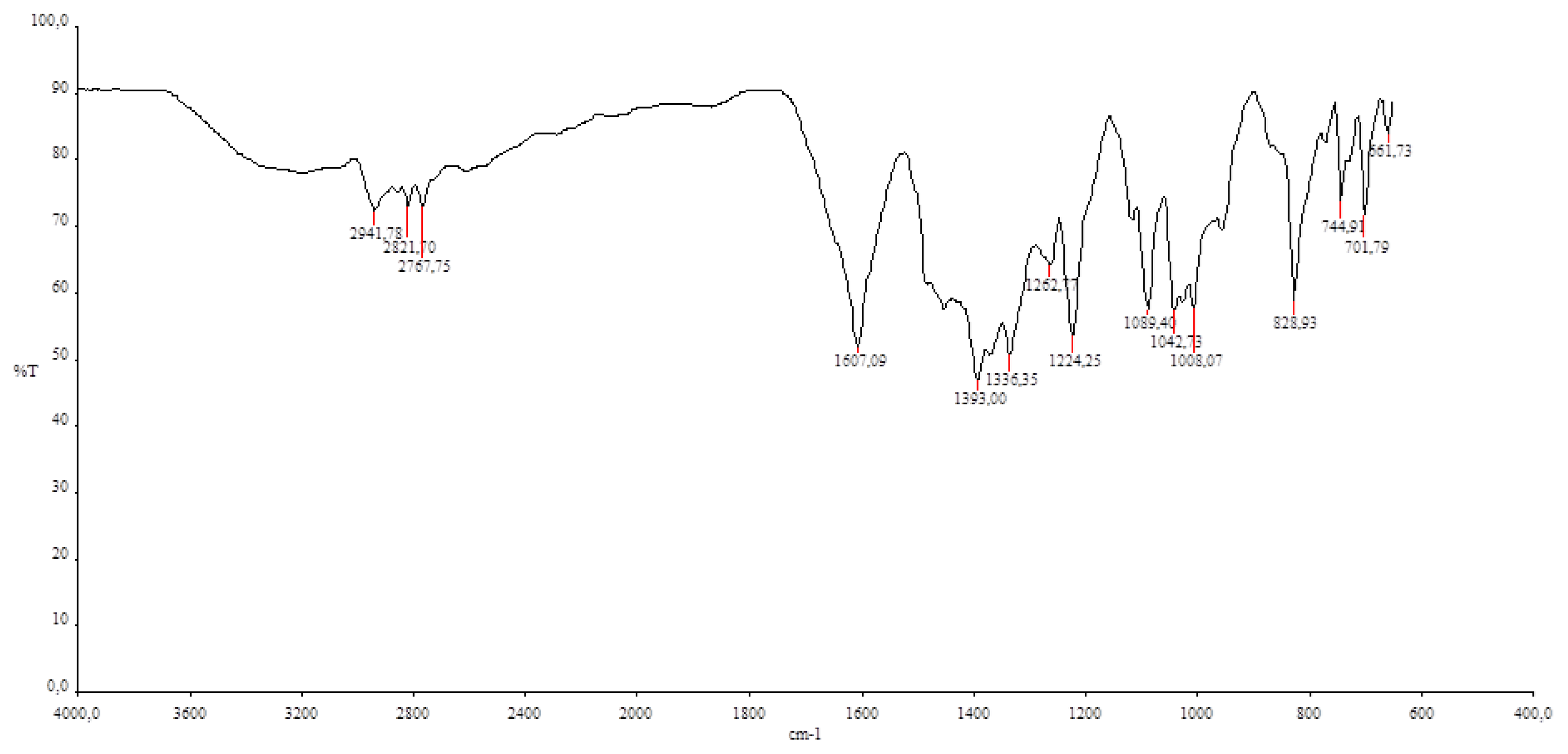

Supplement: Figure S2 — FT-IR spectrum of 4. [file turkjchem-47-5-1160s2.tif]

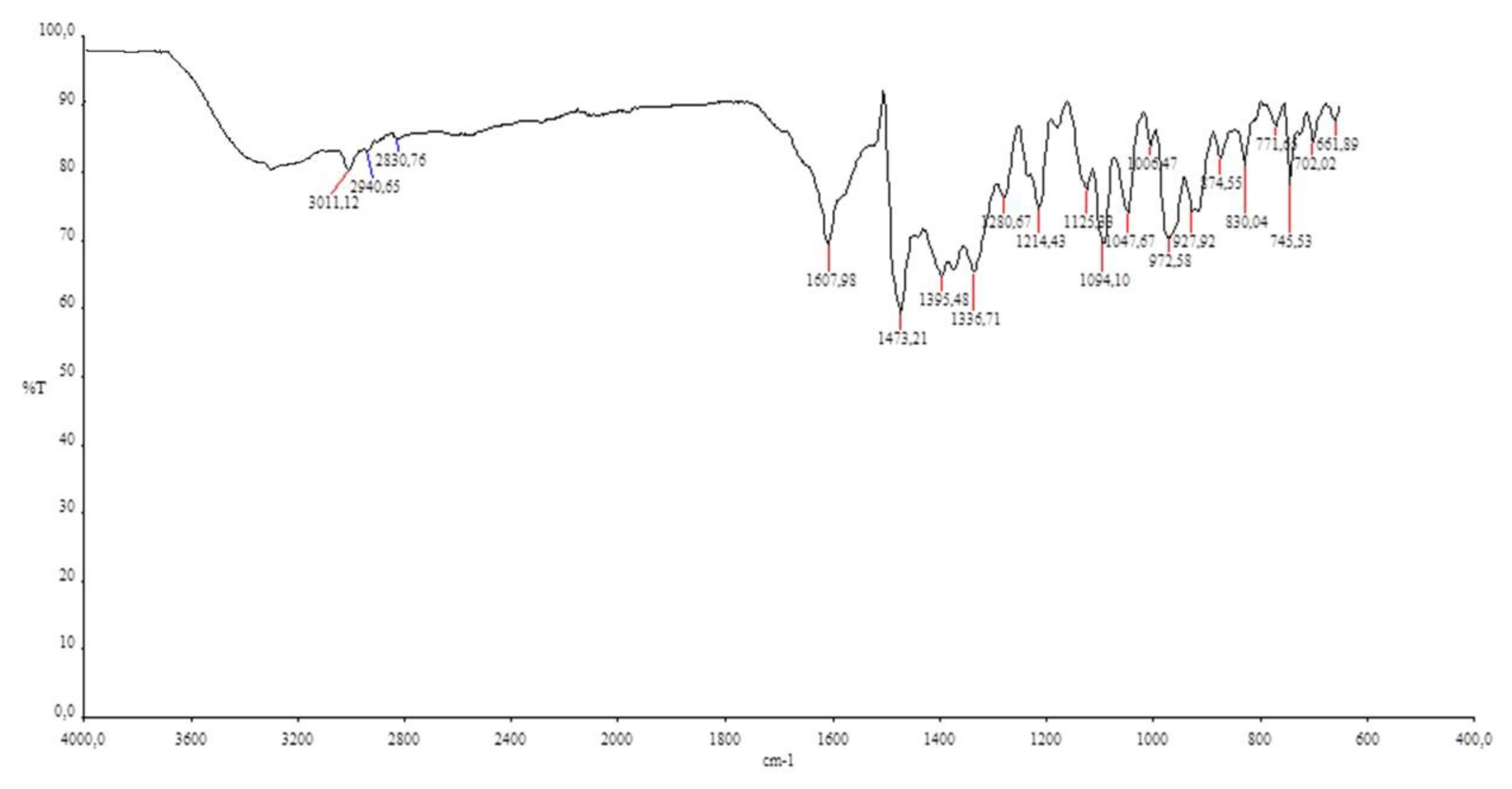

Supplement: Figure S3 — FT-IR spectrum of 5. [file turkjchem-47-5-1160s3.tif]

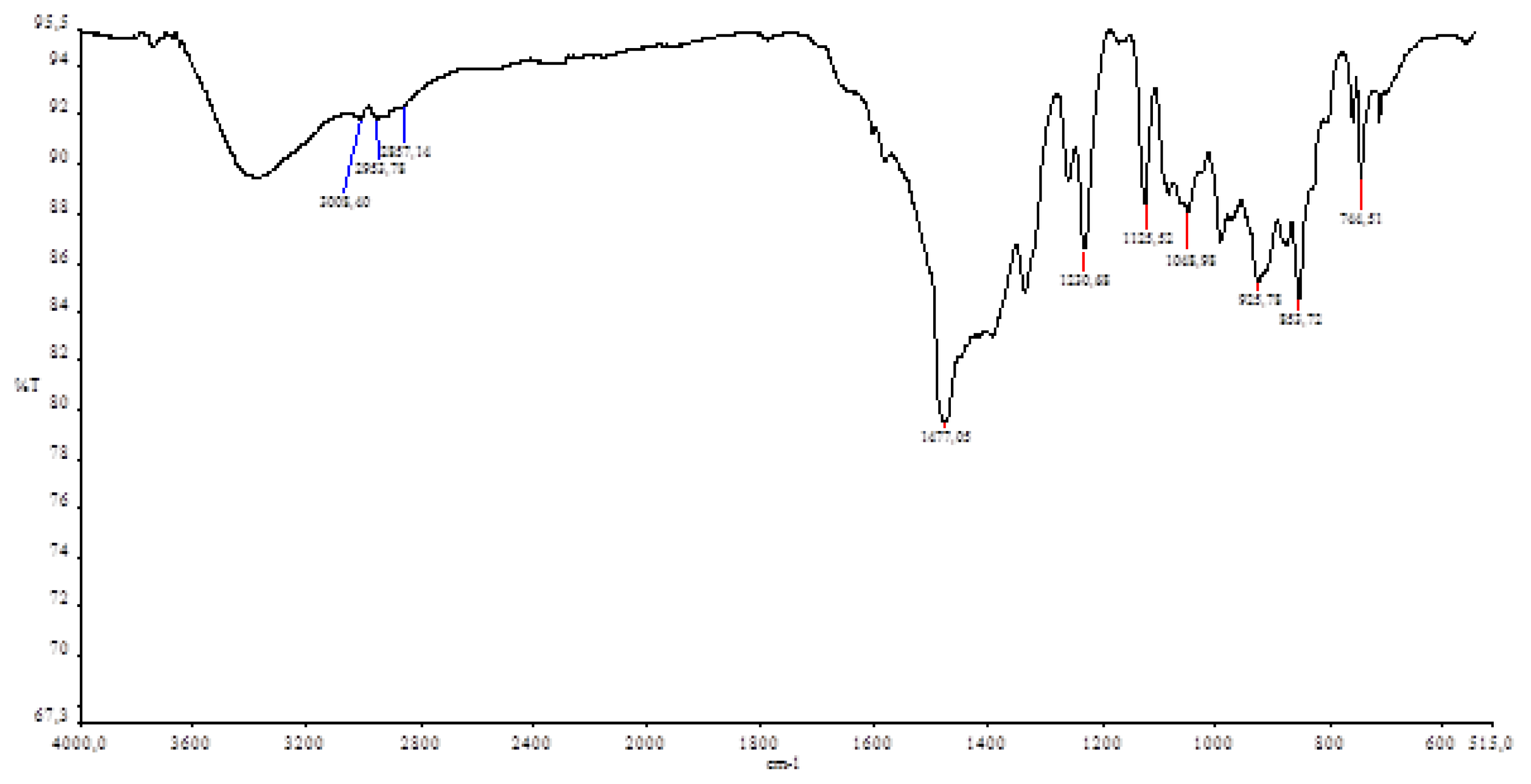

Supplement: Figure S4 — FT-IR spectrum of 7. [file turkjchem-47-5-1160s4.tif]

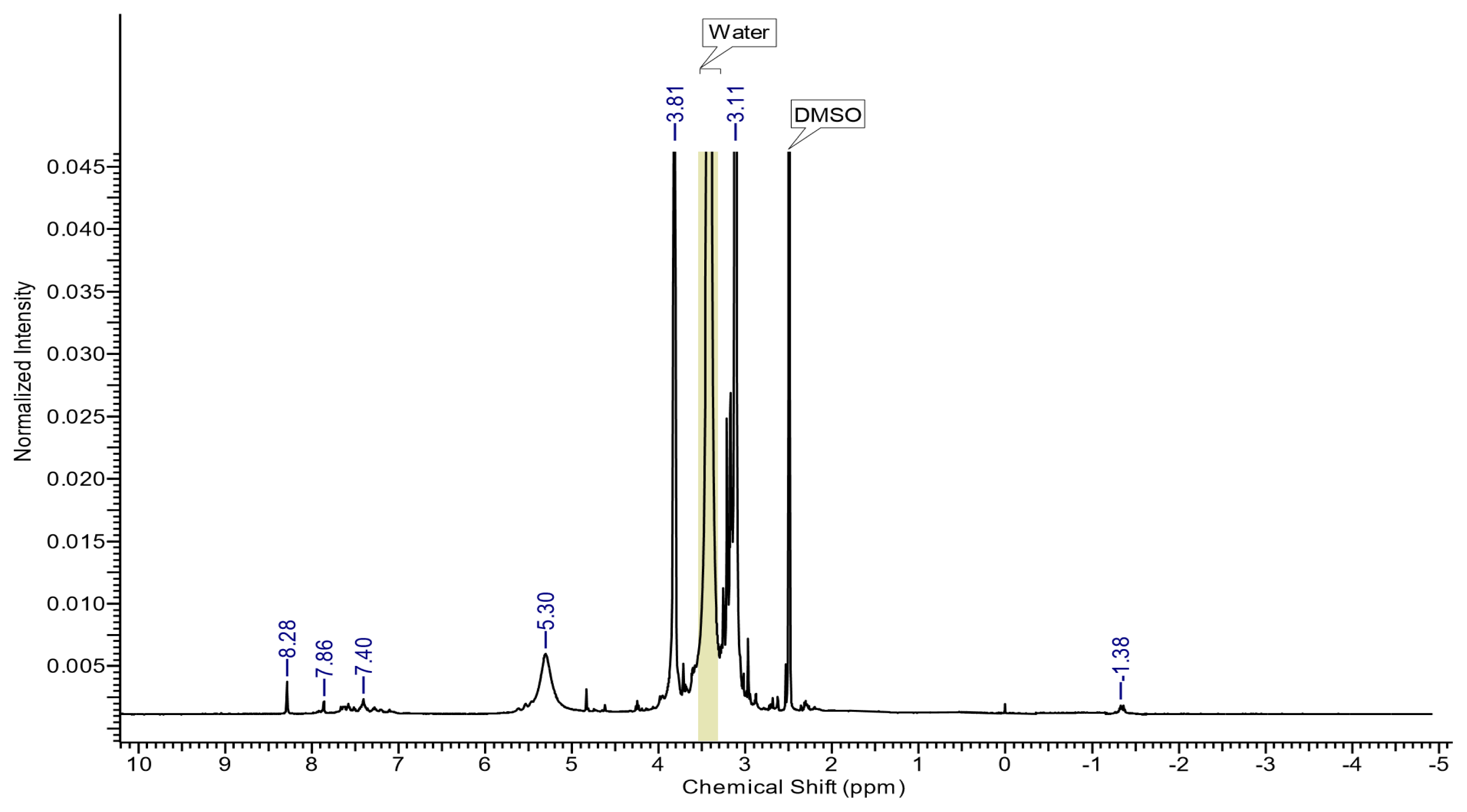

Supplement: Figure S5 — 1H NMR spectrum of 2 in d6-DMSO. [file turkjchem-47-5-1160s5.tif]

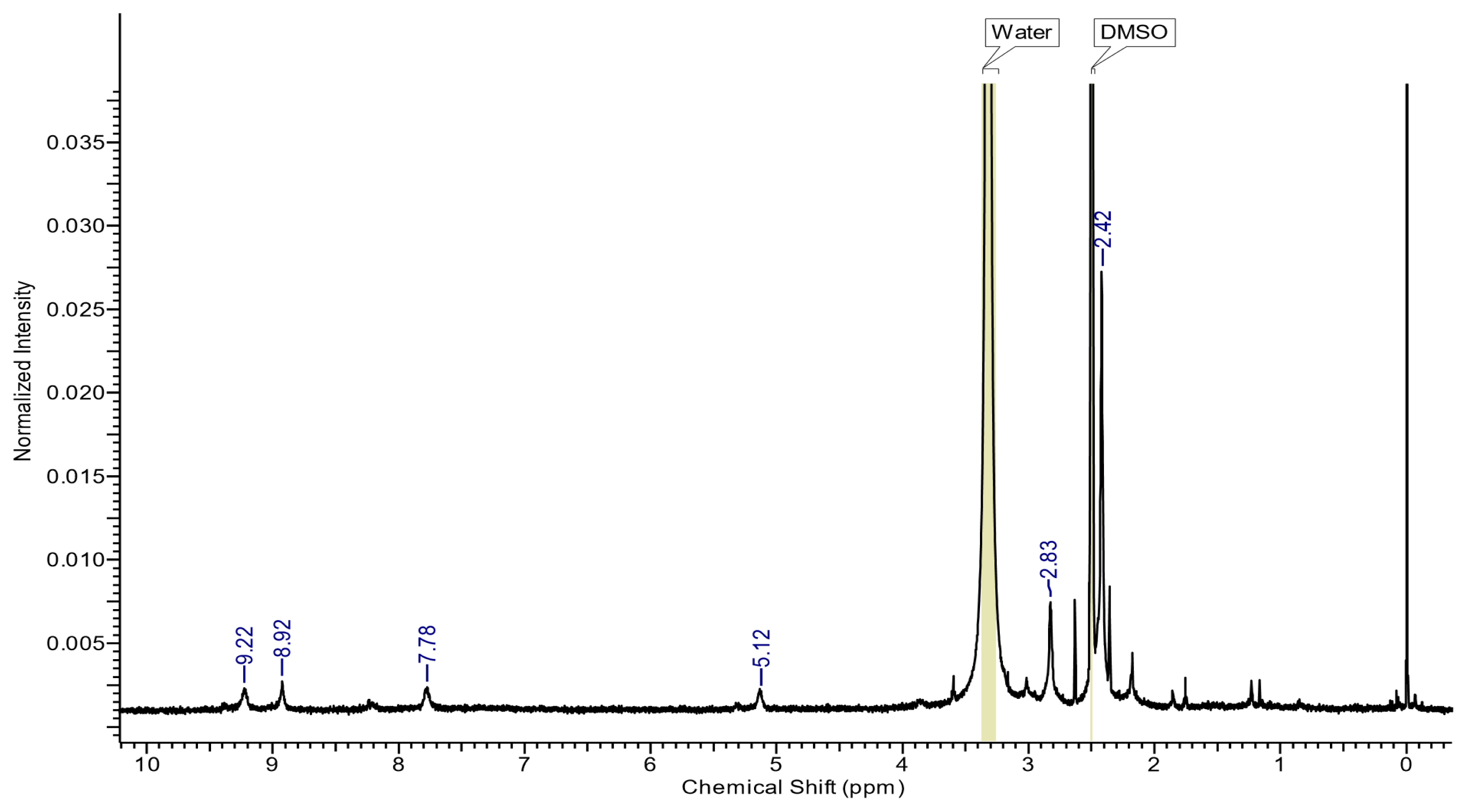

Supplement: Figure S6 — 1H NMR spectrum of 4 in d6-DMSO. [file turkjchem-47-5-1160s6.tif]

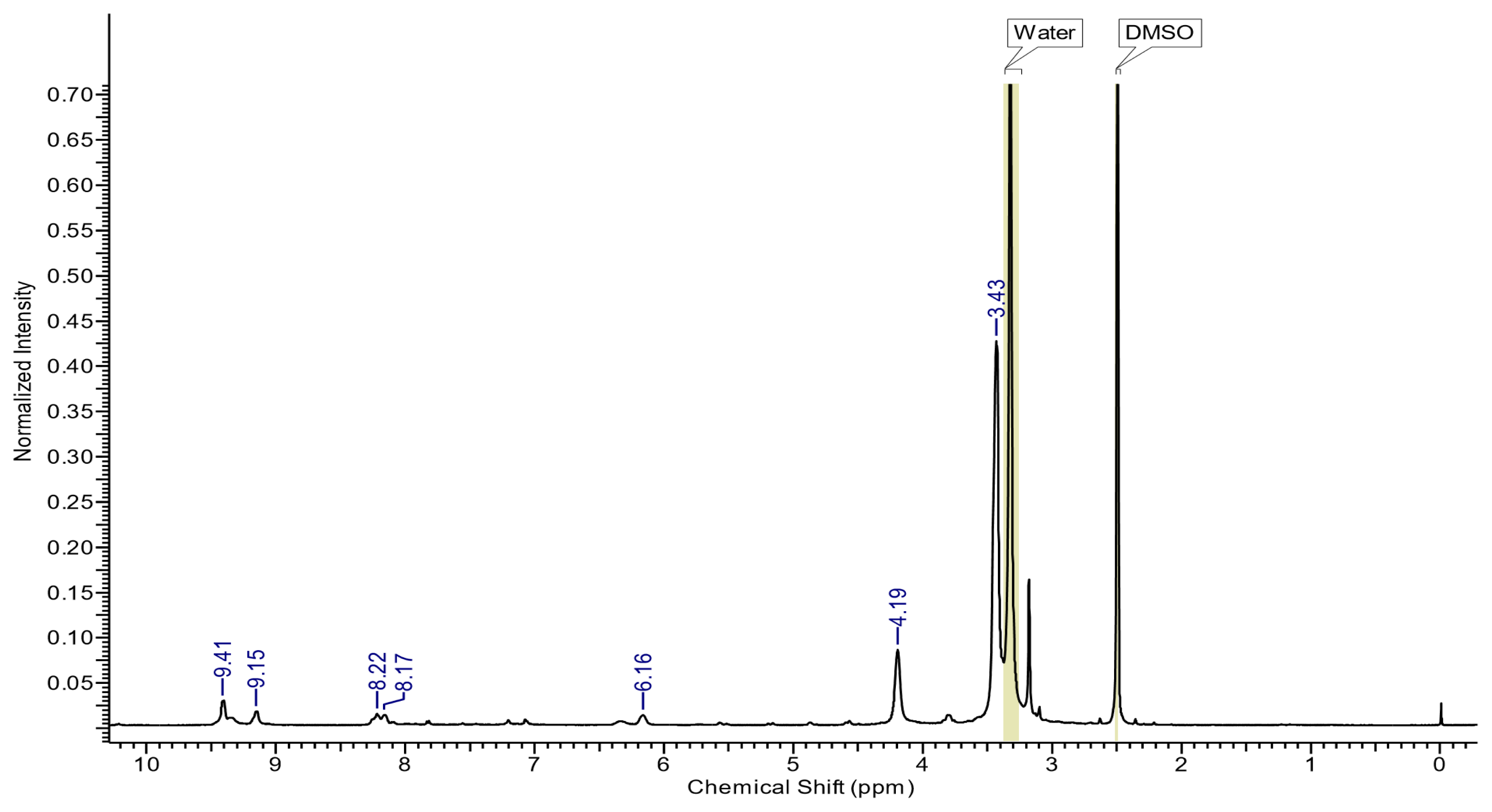

Supplement: Figure S7 — 1H NMR spectrum of 7 in d6-DMSO. [file turkjchem-47-5-1160s7.tif]

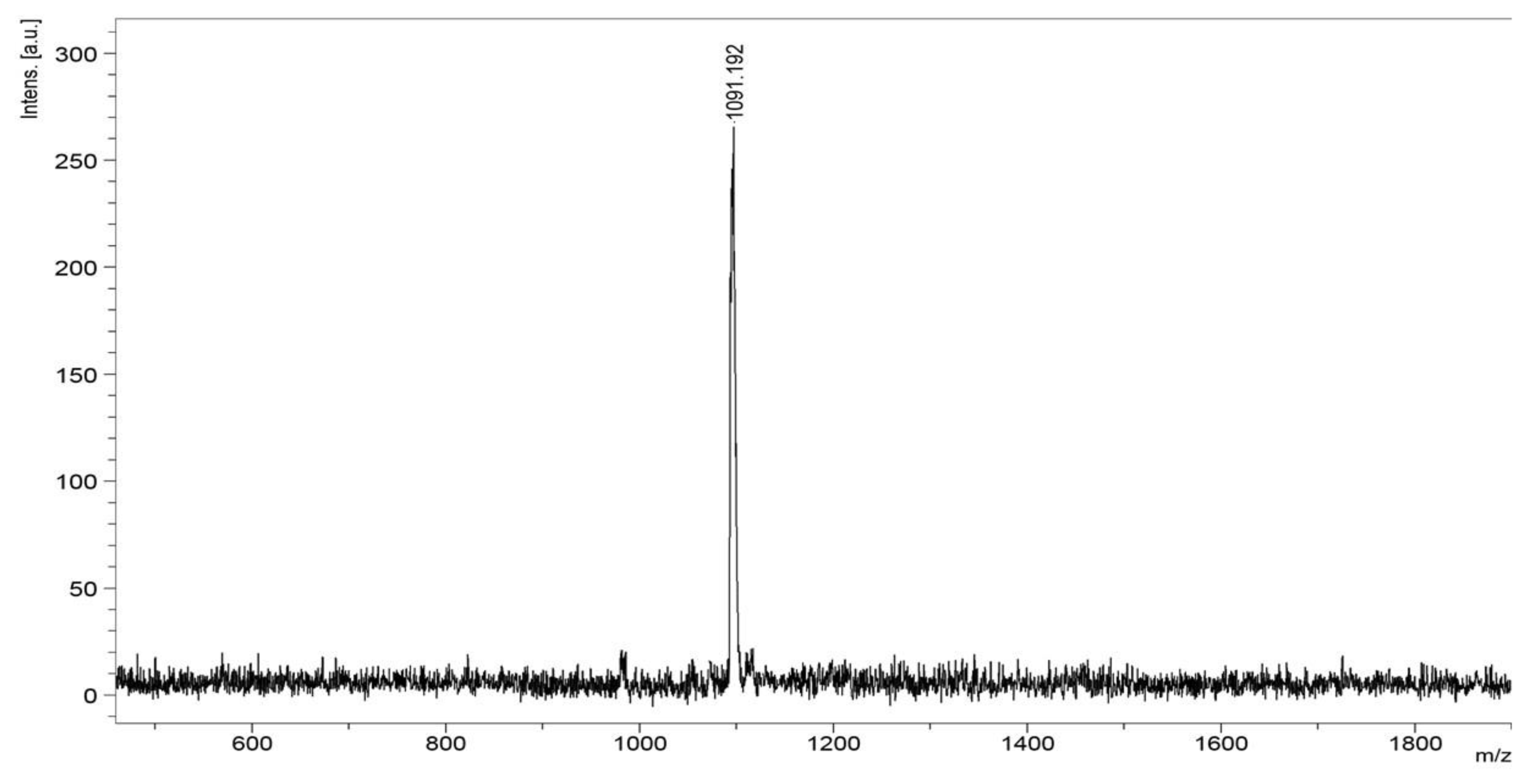

Supplement: Figure S8 — MALDI-TOF MS spectrum of 2. [file turkjchem-47-5-1160s8.tif]

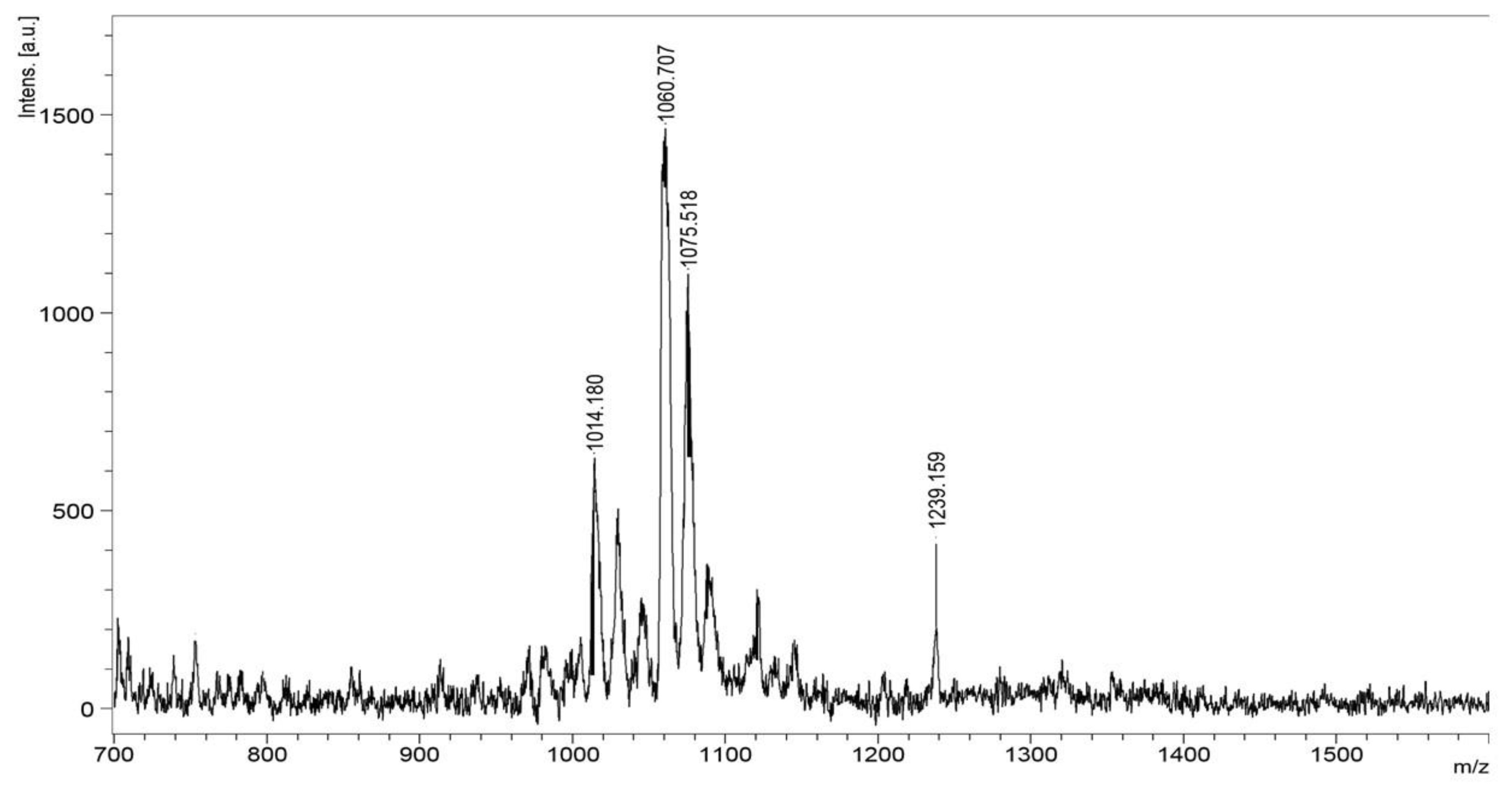

Supplement: Figure S9 — MALDI-TOF MS spectrum of 4. [file turkjchem-47-5-1160s9.tif]

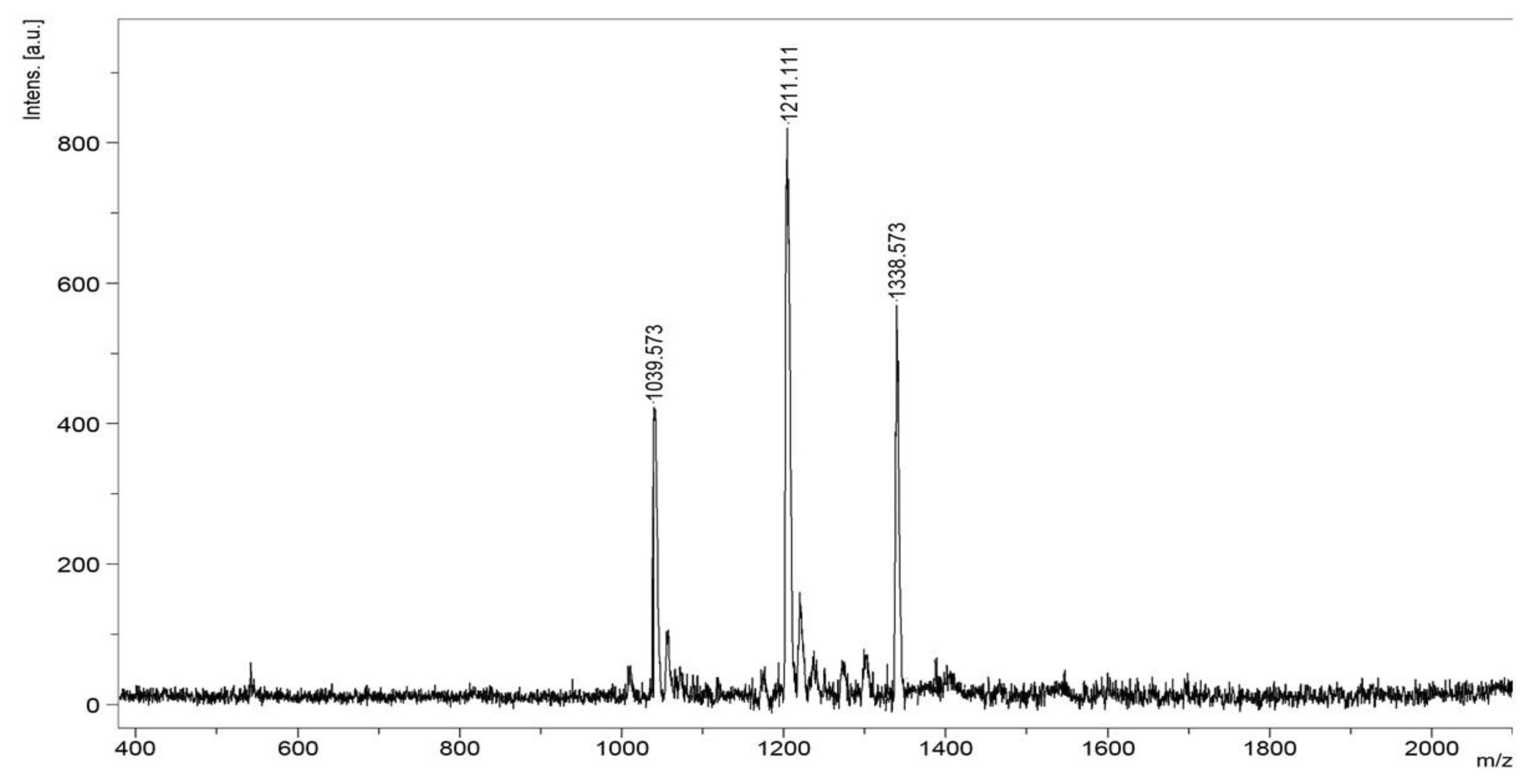

Supplement: Figure S10 — MALDI-TOF MS spectrum of 5. [file turkjchem-47-5-1160s10.tif]

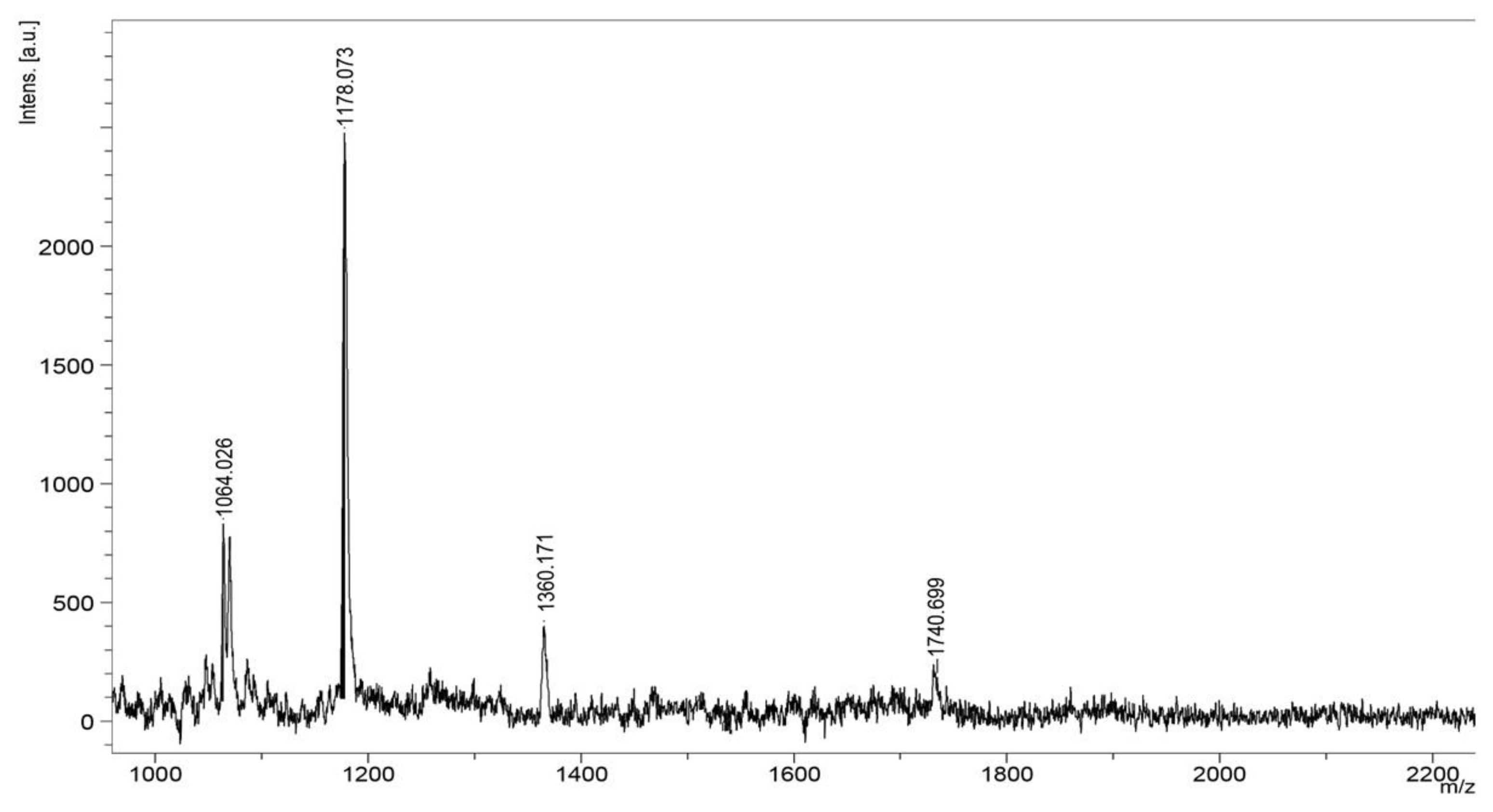

Supplement: Figure S11 — MALDI-TOF MS spectrum of 7. [file turkjchem-47-5-1160s11.tif]
